# Genotypic Characterization of Methicillin-Resistant *Staphylococcus aureus* Recovered at Baseline from Phase 3 Pneumonia Clinical Trials for Ceftobiprole

**DOI:** 10.1089/mdr.2014.0307

**Published:** 2016-01-01

**Authors:** Rodrigo E. Mendes, Lalitagauri M. Deshpande, Andrew J. Costello, David J. Farrell, Ronald N. Jones, Robert K. Flamm

**Affiliations:** JMI Laboratories, North Liberty, Iowa.

## Abstract

Baseline methicillin-resistant *Staphylococcus aureus* (MRSA) isolates from patients with nosocomial and community-acquired pneumonia collected during Phase 3 trials for ceftobiprole were characterized. Eighty-four unique isolates from patients enrolled in Europe (50.0%), Asia-Western Pacific region (APAC; 20.2%), North America (19.0%), Latin America (8.3%), and South Africa (2.4%) were included. Antimicrobial susceptibility testing was performed by broth microdilution and isolates screened for Panton-Valentine leukocidin. SCC*mec* and *agr* types were determined. Strains were subjected to pulsed-field gel electrophoresis and *spa* typing. Clonal complexes (CCs) were assigned based on *spa* and/or multilocus sequence typing. Most isolates were CC5-MRSA-I/II/IV (44.0%; 37/84), followed by CC8-MRSA-IV (22.6%; 19/84) and CC239-MRSA-III (21.4%; 18/84). Other MRSA formed seven clonal clusters. Isolates from North America were associated with USA100, while those from South America belonged to the Cordobes/Chilean CC. A greater clonal diversity was observed in Europe; however, each country had CC5, CC8, or CC239 as prevalent lineages. Isolates from APAC were CC5-MRSA-II (47.1%; 8/17) or CC239-MRSA-III (47.1%; 8/17). Isolates carrying SCC*mec* I and III had ceftobiprole MIC_50_ values of 2 μg/ml, while those isolates with SCC*mec* II and IV had MIC_50_ values of 1 μg/ml. Ceftobiprole inhibited 96% and 100.0% of the isolates at ≤2 and ≤4 μg/ml, respectively. These isolates represented common circulating MRSA clones. Ceftobiprole demonstrated *in vitro* activity with a slight variation of minimum inhibitory concentrations (MICs) according to SCC*mec* or clonal type.

## Introduction

*S**taphylococcus aureus*, including methicillin-resistant isolates (MRSA), remains a leading cause of human bacterial infections in the European Union (EU), USA, and other parts of the world.^15,18,19^ Moreover, MRSA infections account for 44% of hospital-acquired infections (HAI) among institutions of the EU member States, Iceland, and Norway.^19^ Recent reports have demonstrated that the incidence of invasive diseases caused by MRSA has been in decline in England,^21^ as well as in USA^6,16,17,37^ and Canada.^39^ These changes in the incidence of MRSA infections are still poorly understood; but it is clear that the epidemiology of MRSA causing community-acquired infections and HAI continues to evolve in the Americas, Europe, and elsewhere.^1,2,4,9,11,13,32^

The usual high rates of MRSA infections and the attributable mortality and costs associated with these infections have prompted the development of new anti-gram-positive agents, including ceftobiprole.^19^ Ceftobiprole is a novel and broad-spectrum cephalosporin for intravenous administration. This agent has demonstrated an anti-MRSA activity due to its high affinity for the *S. aureus* penicillin-binding protein 2a (PBP2a) as well as the normal complement of β-lactam-sensitive PBPs. Ceftobiprole has also shown *in vitro* activity against the common bacterial pathogens causing pneumonia, including *Streptococcus pneumoniae*, *Moraxella catarrhalis*, *Haemophilus influenzae*, and noncarbapenemase expressing extended-spectrum β-lactamase-negative *Klebsiella pneumoniae and Pseudomonas* spp.^5,14,38^

In Phase 3 trials for skin and soft tissue infections, ceftobiprole demonstrated noninferiority compared to vancomycin.^29,30^ Ceftobiprole has also proven noninferiority to ceftriaxone with or without linezolid for the treatment of community-acquired pneumonia (CAP) requiring hospitalization in Phase 3 trials, with overall cure rates in the clinically evaluable population of 86.6% for ceftobiprole and 87.4% for the comparator agents.^28^ In a Phase 3 trial for the treatment of nosocomial pneumonia (NP), ceftobiprole achieved noninferiority when compared to ceftazidime plus linezolid (cure in clinically evaluable patients of 77% for ceftobiprole and 76% for combination therapy). In this same study, ceftobiprole was not as effective as ceftazidime in the subgroup of patients with ventilator-associated pneumonia (VAP).^3^

The objectives of this study were to characterize the MRSA isolates responsible for NP and CAP infections collected during Phase 3 clinical trials for ceftobiprole.

## Materials and Methods

### Clinical isolates

A total of 121 *S. aureus* isolates from 91 subjects collected during the pneumonia clinical trials were forwarded to JMI Laboratories for further characterization. These isolates were part of the study numbers BAP00248/307 (hospital-acquired pneumonia) (119 strains) and from CAP-3001 (hospitalized patients with CAP) (two strains), and were recovered between July 2005 and April 2007. Twenty of the patients had multiple isolates, but only one isolate per patient was included in the analysis presented here and these strains were all recovered at the first study visit, except for five strains collected during follow-up study visits (1–3 days after study enrollment). Finally, 84 (54 and 28 from NP and VAP infections, respectively) and two (CAP) isolates from studies BAP00248/307 and CAP-3001, respectively, were part of the analysis. The isolates included in this study were recovered from hospitalized patients in Europe (42/84; 50.0%), Asia-Western Pacific region (17/84; 20.2%), North America (16/84; 19.0%), Latin America (7/84; 8.3%), and South Africa (2/84; 2.4%).

### SCC*mec* typing and detection of PVL genes

SCC*mec* types (I through VI) were characterized using a multiplex polymerase chain reaction (PCR) strategy.^25,31^ Panton-Valentine leukocidin (PVL) (*lukF-PV* and *lukS-PV*) screening was performed by using multiplex real-time (RT)-PCR assays, as previously described.^20^

### Epidemiologic typing of MRSA

Chromosomal DNA was subjected to pulsed-field gel electrophoresis (PFGE) after digestion with *Sma*I.^22^ Gel pattern analysis was performed using the GelCompar II software (Applied Math) and the patterns obtained compared to those of the major USA and international clones, which were provided by the Network on Antimicrobial Resistance in *S. aureus* (NARSA, www.narsa.net). All strains were subjected to *agr* and *spa* typing.^34,36^ Clonal complexes (CCs) were assigned based on the *spa* and/or multilocus sequence typing (MLST) results.^24,33^ MRSA strains with *spa* typing results previously associated with specific MLST in the MLST-mapping database (http://spa.ridom.de/mlst) or peer-reviewed publications had the CCs assigned accordingly.^24^ Strains with new *spa* typing denominations and unknown MLST associations, but clustering within PFGE types containing strains with known CC results, were assigned the same CCs. MLST was performed in a given strain when showing *spa* type with unknown MLST association and a unique PFGE type.

### Susceptibility testing

Isolates were tested for susceptibility at a central laboratory facility by broth microdilution according to the Clinical and Laboratory Standards Institute (CLSI) M07-A9 document.^7^ Validation of the minimum inhibitory concentration (MIC) values was performed by concurrent testing of CLSI-recommended quality control reference strains (*S. aureus* ATCC 29213, and *Enterococcus faecalis* ATCC 29212).^8^ Interpretive breakpoints (susceptible ≤2 μg/ml and resistant >2 μg/ml) utilized for ceftobiprole when tested against *S. aureus* were as approved by the European Committee on Antimicrobial Susceptibility Testing^12^ and described in the Zevtera^®^ Summary of Product Characteristics^23^; and the pharmacokinetics/pharmacodynamics (PK/PD) breakpoint (susceptible ≤4 μg/ml and resistant >4 μg/ml) established based on 500 mg administered as a 2-hr intravenous infusion every 8 hr.^27^

## Results and Discussions

Table 1 lists the overall distribution of MRSA clones detected in this study and the majority of isolates were CC5-MRSA-I/II/IV (44.0%; 37/84), which were followed by CC8-MRSA-IV (22.6%; 19/84) and CC239-MRSA-III (21.4%; 18/84). The remaining isolates formed seven clusters with one to three isolates per clonal type. The majority (68.8%; 11/16) of clinical trial strains collected from North America were CC5-MRSA-II/IV and *agr* 2 (Table 2). These CC5-MRSA-II/IV isolates grouped within the PFGE NA-D or -G, and the former pattern matched that of USA100 or Canadian MRSA-2.^22^ A single CC5-MRSA-IV isolate from USA also clustering within NA-D harbored a SCC*mec* type IV, which has been designated as the pediatric clone.^26^ Three CC8-MRSA-IV isolates from three different sites harbored *agr* operon type 1 and were PVL-positive. These strains demonstrated PFGE profiles (NA-C) that matched that of USA300,^22^ and one isolate was obtained from a CAP infection. One strain each of CC45-MRSA-II and CC12-MRSA-IV was detected in subjects from USA. CC45-MRSA-II displayed a unique PFGE pattern (NA-B), which matched that of USA600 (Table 2).^22^

**Table 1.** Overall Molecular Characteristics of MRSA Isolates (Unique Strains) Recovered from Patients Enrolled in the Pneumonia Clinical Trials

*Clonal complex*	agr *type*	*SCC*mec *type*	*PVL*	*No. (% of total)*
CC5	2	I/II/IV	−	37 (44.0)
CC5-MRSA-II	2	II	−	26 (31.0)
CC5-MRSA-I	2	I	−	10 (11.9)
CC5-MRSA-IV	2	IV	−	1 (1.2)
CC8^a^	1	IV	−/+	19 (22.6)
CC239^b^	1	III	−	18 (21.4)
CC30	3	II	−	3 (3.6)
CC22	1	IV	−	2 (2.4)
CC1	3	IV	−	1 (1.2)
CC12	2	IV	−	1 (1.2)
CC45	1	II	−	1 (1.2)
CC72	1	IV	−	1 (1.2)
CC80	3	IV	+	1 (1.2)

^a^ Three PVL-positive strains from the United States and displaying PFGE patterns similar to USA300. One isolate was associated with CAP.

^b^ One isolate was associated with CAP.

CC, Clonal complex; CAP, community-acquired pneumonia; PFGE, pulsed-field gel electrophoresis; PVL, Panton-Valentine leukocidin.

**Table 2.** Overall Epidemiologic Data of Unique MRSA Isolates Recovered During the Pneumonia Clinical Trials

*Region/country*^a^*(no. tested)*		*CC*	*No. (%)*^b^	*SCC*mec	*PVL*	agr	*PFGE*	spa
NA (16)	USA (14)	45	1 (6.3)	II	−	1	NA-B^c^	t004
		8	3 (18.8)	IV	+	1	NA-C^d^	t008
		5	8 (50.0)	II/IV	−	2	NA-D^e^	t002/t242
		12	1 (6.3)	IV	−	2	NA-E	t160
		5	1 (6.3)	II	−	2	NA-G	t002
	Canada (2)	5	2 (12.5)	II	−	2	NA-D^e^	t002
EU (42)	Germany (3)	5	2 (4.8)	II	−	2	EU-G^e^	t003
		22	1 (2.4)	IV	−	1	EU-Q^f^	t904
	Hungary (7)	5	3 (7.1)	II	−	2	EU-G^e^	t002
		5	1 (2.4)	II	−	2	EU-H	t002
		5	3 (7.1)	I	−	2	EU-N	t041
	Israel (1)	5	1 (2.4)	II	−	2	EU-G^e^	t002
	Lithuania (1)	8	1 (2.4)	IV	−	1	EU-F	t008
	Romania (10)	1	1 (2.4)	IV	−	3	EU-A^g^	t127
		239	4 (9.5)	III	−	1	EU-D	t030
		239	4 (9.5)	III	−	1	EU-E	t030
		80	1 (2.4)	IV	+	3	EU-S	t044
	Russia (14)	8	1 (2.4)	IV	−	1	EU-I	t008
		8	1 (2.4)	IV	−	1	EU-K	t008
		8	1 (2.4)	IV	−	1	EU-L	t008
		8	11 (26.2)	IV	−	1	EU-M	t008/t2032
	Serbia and Montenegro (3)	239	2 (4.8)	III	−	1	EU-E	t030, t632
		5	1 (2.4)	I	−	2	EU-N	t041
	Spain (1)	22	1 (2.4)	IV	−	1	EU-Q^f^	t717
	Ukraine (1)	8	1 (2.4)	IV	−	1	EU-J	t008
	United Kingdom (1)	30	1 (2.4)	II	−	3	EU-P^h^	t012
APAC (17)	Australia (1)	239	1 (5.9)	III	−	1	AS-A	t037
	China (3)	239	3 (17.6)	III	−	1	AS-D	t030
	Korea (8)	5	2 (11.8)	II	−	2	AS-C	t002, t6267
		5	5 (11.8)	II	−	2	AS-E	t2060
		72	1 (5.9)	IV	−	1	AS-F^i^	t148
	Taiwan (3)	239	1 (5.9)	III	−	1	AS-A^j^	t037
		239	1 (5.9)	III	−	1	AS-B	t037
		5	1 (5.9)	II	−	2	AS-C	t214
	Thailand (2)	239	2 (11.8)	III	−	1	AS-B	t037, t654
LA (7)	Argentina (6)	5	4 (57.1)	I	−	2	LA-B^k^	t149
		5	2 (28.6)	I	−	2	LA-C	t149
	Brazil (1)	5	1 (14.3)	II	−	2	LA-A^e^	t002
SA (2)	South Africa (2)	30	2 (100.0)	II	−	3	AF-A^h^	t012

^a^ APAC, Asia-Pacific region; EU, Europe (including Israel); SA, South Africa; NA, North America; LA, Latin America.

^b^ Percentage within each region.

^c^ PFGE profile similar to USA600.

^d^ PFGE profile similar to USA300. One isolate was associated with CAP.

^e^ PFGE profile similar to USA100 or Pediatric clone (NA-D, CC5-MRSA-IV).

^f^ PFGE profile similar to UK-eMRSA-15.

^g^ PFGE profile similar to USA400.

^h^ PFGE profile similar to USA200.

^i^ PFGE profile similar to USA700.

^j^ One isolate was associated with CAP.

^k^ PFGE profile similar to the Cordobes/Chilean clone.

MRSA isolates from Europe grouped within seven CCs. Most isolates (37/42; 88.1%) were CC8-MRSA-IV (38.1%; 16/42), CC239-MRSA-III (23.8%; 10/42), or CC5-MRSA-I/II (26.2%; 11/42; Table 2). Of note, CC8-MRSA-IV (*agr* 1) isolates were mostly from Russia (87.5%; 14/16) and one strain each from Ukraine and Lithuania. These strains clustered within six PFGE types belonging to a single *spa* type (t008), except for one strain having a t008 variant (t2032) (Table 2). Isolates belonging to CC239-MRSA-III (PFGE types EU-D and -E), also known as the Brazilian/Hungarian clone,^26^ were observed in Romania (80.0%; 8/10) or Serbia and Montenegro (20.0%; 2/10), while CC5-MRSA-II strains (t002/t003; *agr* 2) were detected in Germany (two strains), Hungary (four strains), and Israel (one strain). Other minor clones in Europe were CC22 (4.8%; 2/42), CC1 (2.4%; 1/422), CC30 (2.4%; 1/422), and CC80 (2.4%; 1/422; Table 2).

Overall, strains from the APAC region were either CC5-MRSA-II (47.1%; 8/17) or CC239-MRSA-III (47.1%; 8/17), except for a single strain from Korea, which was CC8-MRSA-IV (Table 2). CC5 strains were collected from Korea, except for one strain from Taiwan, whereas CC239 was noted from Australia (one isolate), Taiwan (two isolates), Thailand (two isolates), and China (three isolates). One CC239 isolate was associated with CAP. All isolates recovered from Latin America were CC5-MRSA-I (85.7%; 6/7) or -II (14.3%; 1/7). CC5-MRSA-I strains (Argentina) belonged to PFGE LA-B or -C and the LA-B profile was associated with the prevalent Cordobes/Chilean clone.^35^ A single CC5-MRSA-II (Brazil) strain displayed a PFGE pattern similar to that of the USA100 prototype (Table 2).^22^ Two baseline isolates recovered from a subject in South Africa were CC30-MRSA-II (*agr* 3) and showed a PFGE profile similar to the isolate from the United Kingdom (USA200 or UK-eMRSA-16) described in Table 2.^22^

Table 3 shows the distribution of each clonal type between study arms. Overall, there were only minor differences noted between treatment arms (Table 3). One exception was observed for the CC239-MRSA-III lineage, which was more common in the comparator drug arm (25.5%) than in the ceftobiprole treatment arm (16.2%). However, the difference was not statistically significant (*p-*value (0.107) (Table 3). Ceftobiprole inhibited 96.4% of baseline MRSA isolates at the breakpoint for susceptibility (*i.e.*, ≤2 μg/ml) and showed MIC_50_ and MIC_90_ values of 1 and 2 μg/ml, respectively. Moreover, higher ceftobiprole MIC_50_ values (*i.e.*, 2 μg/ml) were observed for isolates carrying SCC*mec* I and III, while those MRSA with SCC*mec* II and IV had MIC_50_ values of 1 μg/ml (Table 4). Similar observations were previously reported by Farrell *et al.*^14^ and Davies *et al.*^10^

**Table 3.** Distribution of MRSA Lineages Between Pneumonia Clinical Trial Treatment Arms

	*Number of strains (%) by study arm*			
*Clonal complex*	*Ceftobiprole*	*Comparator*^a^	*No. (% of total)*	*OR*^b^
CC5	17 (45.9)	20 (42.6)	37 (44.0)	1.1 (0.5–2.7)
CC8	9 (24.3)	10 (21.3)	19 (22.6)	1.2 (0.4–3.3)
CC239	6 (16.2)	12 (25.5)	18 (21.4)	0.56 (0.2–1.7)
CC30	0 (0.0)	3 (6.4)	3 (3.6)	NC
CC22	1 (2.7)	1 (2.1)	2 (2.4)	NC
CC1	1 (2.7)	0 (0.0)	1 (1.2)	NC
CC12	0 (0.0)	1 (2.1)	1 (1.2)	NC
CC45	1 (2.7)	0 (0.0)	1 (1.2)	NC
CC72	1 (2.7)	0 (0.0)	1 (1.2)	NC
CC80	1 (2.7)	0 (0.0)	1 (1.2)	NC
Total	37 (44.0)	47 (56.0)	84 (100)	NC

^a^ Studies BAP00248 and BAP00307 had linezolid with or without ceftazidime in the comparator arm, while study CAP-3001 had ceftriaxone with or without linezolid.

^b^ Odds ratio and respective 95% CI refer to comparisons of rates for CCs observed between study arms. All *p*-values calculated by χ^2^ test were >0.05.

CI, confidence interval; NC, not calculated.

**Table 4.** Ceftobiprole MIC Distribution When Tested Against Specific SSC*mec* and Clonal Types

	*Number (cumulative%)*^a^*of isolates inhibited at ceftobiprole MIC in μg/ml of:*				*MIC (μg/ml)*	
*Type (no. tested)*	*0.5*	*1*	*2*	*4*	*50%*	*90%*
SCC*mec* I (10)	0 (0.0)	0 (0.0)	**10 (100.0)**		2	2
SCC*mec* II (30)	1 (3.3)	**18 (63.3)**	9 (93.3)	2 (100.0)	1	2
SCC*mec* III (18)	0 (0.0)	1 (5.6)	**16 (94.4)**	1 (100.0)	2	2
SCC*mec* IV (26)	9 (34.6)	**17 (100.0)**			1	1
CC5 (37)^b^	2 (5.4)	16 (48.6)	**17 (94.6)**	2 (100.0)	2	2
CC5-MRSA-I (10)	0 (0.0)	0 (0.0)	**10 (100.0)**		2	2
CC5-MRSA-II (26)	1 (3.8)	**16 (65.4)**	7 (92.3)	2 (100.0)	1	2
CC8 (19)^c^	6 (31.6)	**13 (100.0)**			1	1
CC239 (18)^d^	0 (0.0)	1 (5.6)	**16 (94.4)**	1 (100.0)	2	2
Other (10)	2 (20.0)	**6 (60.0)**	2 (100.0)		1	2
All (84)	10 (11.9)	**36 (54.8)**	35 (96.4)	3 (100.0)	1	2

^a^ Modal MIC values are in bold.

^b^ Ten, 26 and 1 isolates carrying SCC*mec* I, II, and IV, respectively.

^c^ All CC8 isolates carried SCC*mec* IV.

^d^ All CC239 isolates carried SCC*mec* III.

MIC, minimum inhibitory concentration.

The most common clonal types observed among isolates collected from the patients enrolled in the pneumonia Phase 3 clinical trials for ceftobiprole belonged to CC5-MRSA-I/II/IV (44.0%; 37/84), CC8-MRSA-IV (22.6%; 19/84), and CC239-MRSA-III (21.4%; 18/84). Similar results were reported among MRSA baseline isolates collected in a worldwide pneumonia clinical trial for linezolid, where CC5, CC8, and CC239 constituted 56.0, 23.3, and 11.3% of the isolates, respectively.^24^ Of note, although isolates belonging to CC8-MRSA-IV (38.1%) prevailed, the MRSA population in European countries and Israel demonstrated a greater genetic diversity than observed in other regions. The CC8-MRSA-IV lineage seems to be replacing previous commonly detected MRSA clones in this region, such as ST247-MRSA-I (Iberian; CC8), ST228-MRSA-I (South German; CC5), ST239-MRSA-III (CC239; Brazilian/Hungarian), CC22-MRSA-IV (UK-eMRSA-15), and ST45-MRSA-IV (CC45; Berlin). However, one significant study limitation is the relatively low number of baseline pathogens, which precludes a more robust statistical analysis. In summary, the isolates included in the present study were found to represent common clones currently circulating in these study regions and ceftobiprole demonstrated a slight variation in the MIC results according to SCC*mec* or clonal type, but overall inhibited 96.4% and 100.0% of isolates at ≤2 and ≤4 μg/ml, respectively.^27^
